# Differential Effects of a Full and Biased Ghrelin Receptor Agonist in a Mouse Kindling Model

**DOI:** 10.3390/ijms20102480

**Published:** 2019-05-20

**Authors:** An Buckinx, Yana Van Den Herrewegen, Anouk Pierre, Eleonora Cottone, Khoubaib Ben Haj Salah, Jean-Alain Fehrentz, Ron Kooijman, Dimitri De Bundel, Ilse Smolders

**Affiliations:** 1 Department of Pharmaceutical Chemistry, Drug Analysis and Drug Information, Research Group Experimental Pharmacology, Center for Neurosciences (C4N), Vrije Universiteit Brussel (VUB), Laarbeeklaan 103, 1090 Brussels, Belgium; an.buckinx@vub.be (A.B.); Yana.Van.Den.Herrewegen@vub.be (Y.V.D.H.); anouk.pierre@vub.be (A.P.); Eleonora.cottone@vub.be (E.C.); dimitri.de.bundel@vub.be (D.D.B.); 2 Max Mousseron Institute of Biomolecules UMR524, CNRS, University of Montpellier, Ecole Nationale Supérieure de Chimie de Montpellier, 34090 Montpellier, France; khoubaib.bhs@gmail.com (K.B.H.S.); jean-alain.fehrentz@univ-montp1.fr (J.-A.F.); 3 Research Group Experimental Pharmacology, Center for Neurosciences (C4N), Vrije Universiteit Brussel (VUB), 1050 Brussels, Belgium; ron.kooijman@vub.be

**Keywords:** epilepsy, biased signaling, ghrelin receptor, JMV-1843, YIL781, JMV-2959, β-arrestin

## Abstract

The ghrelin system has received substantial recognition as a potential target for novel anti-seizure drugs. Ghrelin receptor (ghrelin-R) signaling is complex, involving Gα_q/11_, Gα_i/o_, Gα_12/13_, and β-arrestin pathways. In this study, we aimed to deepen our understanding regarding signaling pathways downstream the ghrelin-R responsible for mediating anticonvulsive effects in a kindling model. Mice were administered the proconvulsive dopamine 1 receptor-agonist, SKF81297, to gradually induce a kindled state. Prior to every SKF81297 injection, mice were treated with a ghrelin-R full agonist (JMV-1843), a Gα_q_ and Gα_12_ biased ligand unable to recruit β-arrestin (YIL781), a ghrelin-R antagonist (JMV-2959), or saline. Mice treated with JMV-1843 had fewer and less severe seizures compared to saline-treated controls, while mice treated with YIL781 experienced longer and more severe seizures. JMV-2959 treatment did not lead to differences in seizure severity and number. Altogether, these results indicate that the Gα_q_ or Gα_12_ signaling pathways are not responsible for mediating JMV-1843′s anticonvulsive effects and suggest a possible involvement of β-arrestin signaling in the anticonvulsive effects mediated by ghrelin-R modulation.

## 1. Introduction

Epilepsy is a severe neurological condition affecting millions of patients worldwide. It is characterized by episodes of excessive neuronal discharges, so-called seizures, which can be restricted to a focal brain area or engage both hemispheres [1]. Ongoing seizures have detrimental effects on neuronal viability, brain inflammation, and overall quality of life [2,3], rendering seizure suppression essential. However, despite the availability of numerous therapeutics, a staggering 30% of patients is not seizure-free, a proportion that has remained relatively stable for the last decades [4].

The use of neuropeptides in epilepsy received considerable attention; one among them is ghrelin. This 28 amino-acid neuropeptide and its G-protein coupled receptor (GPCR), the ghrelin receptor (ghrelin-R), are best known for growth hormone (GH) release and their orexigenic actions [5,6]. Nonetheless, the ghrelin system exerts a broad spectrum of other critical physiological functions, including synaptic plasticity and excitability [7].

The effect of ghrelin administration on seizures has been investigated preclinically in several studies. Ghrelin attenuated seizure severity in the pentylenetetrazol (PTZ) rat model [8,9], in the intraperitoneal (i.p.) kainic acid mouse model [10], in penicillin-induced seizures in rats [11], and in the rat intrahippocampal pilocarpine model [12]. However, ghrelin was not able to affect seizure severity in the rat i.p. pilocarpine *status epilepticus* model [13,14].

Interestingly, neuronal survival was ameliorated upon ghrelin administration in the rat PTZ model [15], in the lithium pilocarpine model [16], and in the pilocarpine rat model [13,17]. It was also shown to exert anti-inflammatory effects in the kainic acid mouse model [10], and in the rat PTZ model [18]. Additionally, locally infused ghrelin appeared to dose-dependently improve spatial memory in PTZ-treated rats [19], which is interesting in light of co-morbidities associated with epilepsy.

It is for these reasons that ghrelin and ghrelin-R agonists appear to be appealing candidates for target-driven therapeutic approaches achieving seizure control. However, ghrelin-R signal transduction pathways responsible for these anticonvulsive effects are up to now unknown.

Ghrelin-R signaling occurs via two main signaling pathways, Gα_q/11_ and β-arrestin signaling, but also via Gα_i/o_ and Gα_12/13_ signaling [20,21]. When ghrelin binds to its receptor, conformational changes in the ghrelin-R allow the release of the G_βγ_ complex from the G_α_-subunit and activation of the associated second messenger molecules and downstream signaling pathways [21].

Gα_q/11_ stimulates the classical phospholipase C (PLC)–inositol 1,4,5-trisphosphate (IP3) pathway, generating a considerable rise in intracellular calcium. Additionally, Gα_q/11_ signaling activates the mitogen-activated protein kinases (MAPKs) such as extracellular signal-regulated kinases 1 and 2 (ERK1/2), and promotes the activation of serum-response element (SRE) [22]. Gα_12/13_ activation is essential for the induction of SRE, and besides this also promotes the RhoA kinase signaling pathway. Gα_i/o_ inhibits adenylyl cyclase (AC) and lowers cyclic adenosine monophosphate (cAMP) production. The activation and consequent dissociation of the G-proteins allows for β-arrestin to be recruited toward the receptor [21]. β-arrestin interaction initiates desensitization and endocytosis of the receptor. Accordingly, this internalization halts G-protein dependent signaling and enables G-protein independent signaling to commence [23].

Besides employing multiple pathways, ghrelin-R displays a remarkably high constitutive activity with important implications in vivo, both regarding food intake and GH release [24]. It is therefore not surprising that a multitude of synthetic ligands for the ghrelin-R were developed.

JMV-1843 is a highly potent, full agonist of ghrelin-R, which activates the full subset of ghrelin-R described pathways; Gα_q/11_, Gα_i/o_, and Gα_12/13_, which are eventually halted by β-arrestin recruitment and internalization of the receptor [20,25] (Table 1). Interestingly, this compound recently got approved as a medicinal product in the United States and Europe for the diagnosis of GH deficiency in adults [26].

Not all ligands necessarily activate all downstream signaling pathways associated with a GPCR. This phenomenon is referred to as biased signaling, and implies that a restricted number of signaling pathways are employed instead of the full subset, depending on the ligand that binds the receptor [27]. One of these biased agonists recently described for the ghrelin-R is YIL781 [28]. Primarily, this compound was classified as a ghrelin-R antagonist, but recently it was unveiled that this ligand preferentially activates Gα_q/11_ and Gα_12_ and behaves antagonistic or modestly inverse agonistic toward the other pathways [28]. YIL781 does not lead to β-arrestin recruitment and hence does not induce a rapid termination of Gα_q/11_ signaling (Table 1).

JMV-2959 is a neutral antagonist of ghrelin-R and does not affect the high Gα_q/11_, Gα_i/o_, and Gα_12/13_ basal-ligand-independent constitutive activity [29,30]. β-arrestin is only recruited in an agonist-activated ghrelin-R state [21], and therefore JMV-2959 does not lead to β-arrestin recruitment (Table 1).

Ghrelin and JMV-1843 [25] were demonstrated to exert anticonvulsive effects mediated via the ghrelin-R in multiple seizure or epilepsy models [8,9,10,12,14,18,31]. Also, ghrelin-R inverse agonists were found to be anticonvulsive, and ghrelin-R knock-out mice showed higher seizure thresholds [12]. In an attempt to reconcile these observations, we proposed a novel concept suggesting that anticonvulsive effects are the result of ghrelin-R inactivation [12], possibly via β-arrestin-mediated desensitization and termination of G-protein dependent signaling.

To unravel the contribution of specific signaling pathways to the anticonvulsant effect exerted by ghrelin-R agonists, we investigated the effects of a ghrelin-R full agonist (JMV-1843), and a Gα_q_ and Gα_12_ ghrelin-R biased ligand (YIL781) in the dopamine 1 receptor (D1R)-mediated kindling model [32]. Based on YIL781′s preferential activation of Gα_q_ and Gα_12_ signalization and its inability to recruit β-arrestin, we hypothesized that mice treated with YIL781 will be experiencing more seizures compared to controls. The ghrelin-R neutral antagonist JMV-2959 was shown to inhibit SKF81297-induced behaviors that were dependent on ghrelin-R:D1R heterodimeric interactions [33]. Therefore, we decided to evaluate the effects of JMV-2959 in the D1R-mediated kindling model, as this compound has the potential to interfere with SKF81297-induced seizures.

## 2. Results

### 2.1. SKF81297 Induces Hippocampal Discharges Corresponding to Seizure-Like Behavior

**Figure 1:**
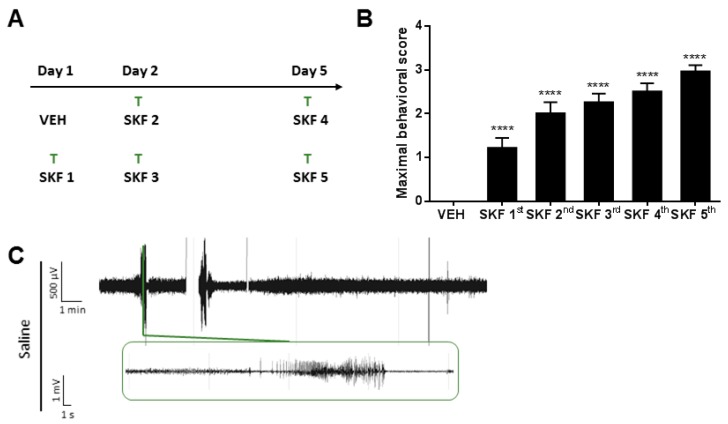
Overview of the D1R-mediated kindling model. (**A**) Experimental design. (**B**) Maximal behavioral score in C57BL/6J mice pooled from the three experiments (*n* = 24). One-way RM ANOVA (Treatment *p* < 0.0001, F (5.000, 115.0) = 31.01). (**C**) Representative trace of saline-treated control mouse during SKF 5th. 0.3 Hz high-pass, 60 Hz low-pass, and 50 Hz power line filters were applied. Data are presented as mean ± SEM. **** *p* < 0.0001. µV, microvolt; mV, millivolt; min, minute; s, second; SKF, SKF81297; T, treatment; VEH, vehicle.

The repeated administration of the D1R agonist SKF81297 induced progressively aggravating seizures, as previously described [32]. In our study, ghrelin-R ligands were administered 30 min prior to an SKF81297 challenge, and consisted of either JMV-1843, YIL781, JMV-2959, or saline (Figure 1A). Saline-treated control mice subjected to the SKF81297 kindling model exhibited seizures ranging from behavioral arrest (Phase I) or myoclonic twitches to severe generalized tonic–clonic manifestations with wild jumping (Phase IV; Figure 1B, all saline-treated control mice pooled together). Generalized seizures (Phase III and IV) corresponded to clear electrographic deep hippocampal seizures (Figure 1C), while behavioral Phase I and Phase II seizures were occasionally, but not always, detected as spikes in hippocampal electro-encephalographic (EEG) recordings. The model is subject to between-batch variability; therefore, the three experiments that were performed have their own set of control animals. However, the control groups from the three experiments were compared and did not differ significantly from each other regarding seizure severity, number of seizures, or seizure duration (Supplementary Figure S1).

### 2.2. The Ghrelin-R Full agonist, JMV-1843, Is Anticonvulsive in the D1R-Mediated Kindling Model

**Figure 2:**
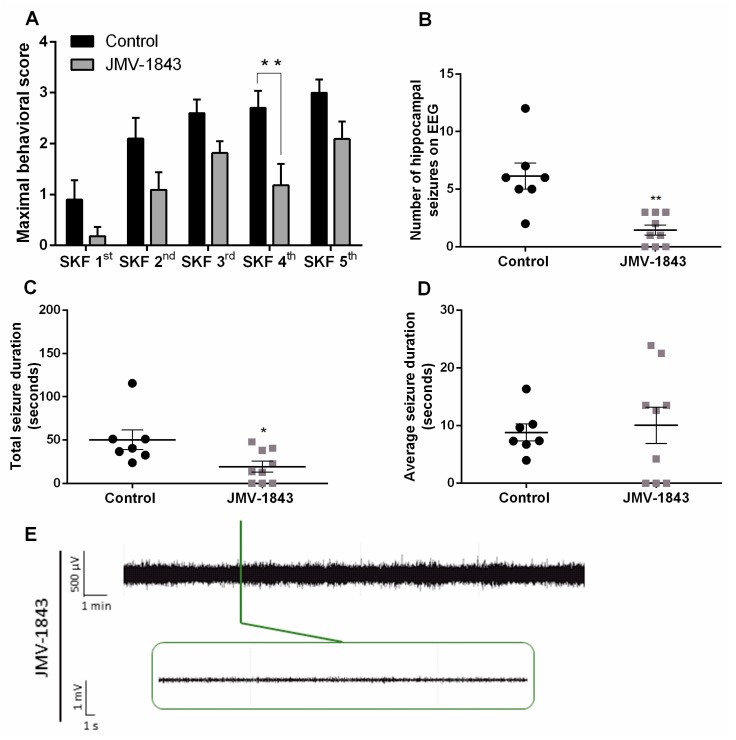
JMV-1843 attenuates seizures in the D1R-mediated kindling model. (**A**) Maximal behavioral score was lower in JMV-1843 treated mice (*n* = 11) compared to saline-treated controls (*n* = 10). Two-way RM ANOVA (Interaction *p* > 0.05, F(4,76) = 0.5059; Time *p* < 0.0001, F(4,76) = 11.87; Treatment *p* < 0.001, F(1,19) = 18.85). (**B**) The number of hippocampal seizures on EEG recordings was lower in JMV-1843 (*n* = 9) treated mice compared to saline-treated controls (*n* = 7). Mann–Whitney test (*p* < 0.01; Mann–Whitney U = 3.500). (**C**) Total seizure duration was lower in JMV-1843 treated mice (*n* = 9) compared to controls (*n* = 7). Mann–Whitney test (*p* < 0.05; Mann–Whitney U = 11.00). (**D**) Average seizure duration did not differ between JMV-1843 treated mice (*n* = 9) and controls (*n* = 7). Mann–Whitney test (*p* > 0.05; Mann–Whitney U = 30.00). (**E**) Representative trace of JMV-1843-treated mouse during SKF 5th. 0.3 Hz high-pass, 60 Hz low-pass, and 50 Hz power line filters were applied. Data are presented as mean ± SEM. * *p* < 0.05; ** *p* < 0.01. EEG, electro-encephalography; µV, microvolt; mV, millivolt; min, minute; s, second; SKF, SKF81297.

**Figure 3:**
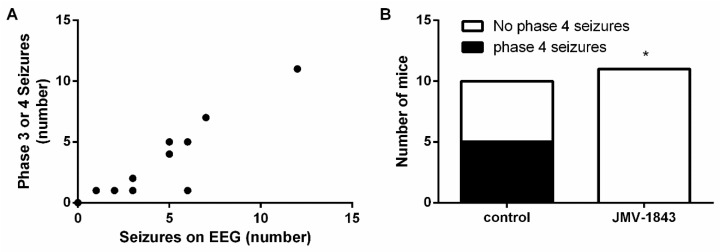
JMV-1843 treatment abolishes Phase IV seizures in the SKF81297-mediated kindling model. (**A**) Correlation between number of Phase III and IV seizures visually observed and number of hippocampal discharges on EEG (controls and JMV-1843-treated mice taken together; *n* = 16 pairs). Pearson correlation (*p* < 0.0001; Pearson’s r = 0.9173). (**B**) 0 % of JMV-1843-treated mice (*n* = 11) experienced Phase IV seizures while 50% of saline-treated controls (*n* = 10) did. Fisher’s exact test (*p* < 0.05). * *p* < 0.05; EEG, electro-encephalography.

In the first experiment, we investigated whether administration of the ghrelin-R full agonist, JMV-1843, was able to modulate seizure activity. JMV-1843 lowered seizure severity (*p* < 0.001 compared to saline-treated controls), represented by maximal behavioral score obtained per SKF81297 challenge (Figure 2A). Behavioral manifestations corresponded to intrahippocampal discharges monitored via EEG (*p* < 0.0001; Figure 3A). As Phase I and II seizures occasionally, but not always, coincided with single spikes, only Phase III and IV generalized seizures were taken into account for quantification of EEG measurements. EEG signals that were contaminated with heart rate due to damaged lead insulation were discarded from EEG analyses, explaining the smaller sample sizes in the groups for EEG analyses compared to behavior. JMV-1843-treated mice experienced an average of 1.44 ± 0.44 hippocampal seizures during the total kindling procedure compared to an average of 6.14 ± 1.14 for control mice (*p* < 0.001; Figure 2B). Correspondingly, total seizure duration was lower in JMV-1843-treated mice (19.44 ± 6.25 s) compared to saline-treated controls (50.19 ± 11.51 s; *p* < 0.05; Figure 2C), while average seizure duration did not differ between both groups (8.79 ± 1.47 s for saline-treated controls versus 10.03 ± 3.15 s for JMV-1843-treated mice; Figure 2D). Total seizure duration is the sum of the duration of all seizures that were experienced during the total kindling procedure. For average seizure duration, total seizure duration was divided by the number of hippocampal seizures that mice experienced during the total kindling procedure. If mice experienced no seizures, this was denoted as an average seizure duration of 0 s. Interestingly, none of the mice treated with JMV-1843 experienced Phase IV seizures, while 50% of saline-treated control mice did (*p* < 0.05; Figure 3B).

All together, these data indicate that JMV-1843 is anticonvulsive in the D1R-mediated kindling model by attenuating seizure severity and decreasing the number of seizures that mice experienced.

### 2.3. The Ghrelin-R Gα_q_ Partial agonist, YIL781, Increases Seizure Burden in the D1R-Mediated Kindling Model

As JMV-1843 is a full agonist of the ghrelin-R and hence activates the full subset of signaling pathways downstream of its receptor, we aimed to further clarify the contribution of Gα_q/11_ signaling in mediating anticonvulsive effects exerted by JMV-1843. YIL781 behaves as a biased agonist toward Gα_q/11_ while it has slightly inverse agonistic properties toward β-arrestin signaling [28]. Therefore, we investigated the effects of YIL781-treatment in the D1R-mediated kindling model.

**Figure 4:**
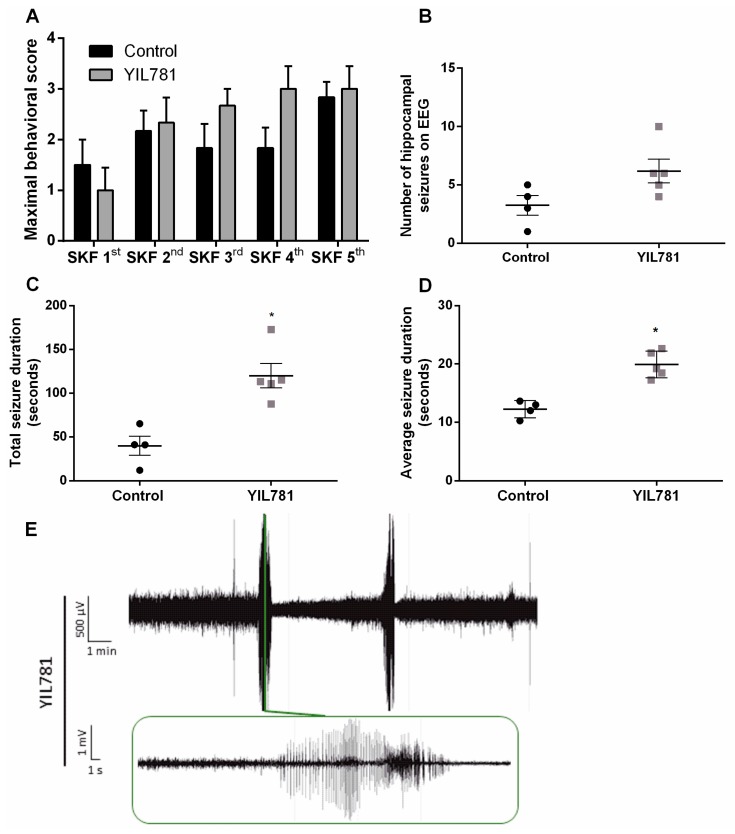
The effect of YIL781 on SKF81297 induced seizures. (**A**) Maximal behavioral scores obtained by YIL781-treated (*n* = 6) and control (*n* = 6) animals. Two-way RM ANOVA (interaction *p* > 0.05, F(4,40) = 1.035; time *p* < 0.05, F(4,40) = 3.597; treatment *p* > 0.05, F(1,10) = 3.071). (**B**) The number of hippocampal seizures on EEG did not differ between YIL781-treated mice (*n* = 4) and saline-treated control mice (*n* = 5). Mann–Whitney test (*p* = 0.056; Mann–Whitney U = 2.000). (**C**) Total seizure duration was higher in YIL781 treated mice (*n* = 5) compared to saline-treated control mice (*n* = 4). Mann–Whitney test (*p* < 0.05; Mann–Whitney U = 0.0). (**D**) YIL781-treated mice (*n* = 5) have higher average seizure durations compared to saline-treated control mice (*n* = 4). Mann–Whitney test (*p* < 0.05; Mann–Whitney U = 0.0). (**E**) Representative traces of YIL781-treated mouse already experiencing several seizures during SKF 3rd. 0.3 Hz high-pass, 60 Hz low-pass, and 50 Hz power line filters were applied. Data are presented as mean ± SEM. *, *p* < 0.05. EEG, electro-encephalography; µV, microvolt; mV, millivolt; min, minute; s, second; SKF, SKF81297.

Maximal behavioral score obtained per SKF81297 challenge did not differ between saline-treated control animals and YIL781-treated animals (Figure 4A). EEG analyses revealed that YIL781-treated mice had an average of 6.2 ± 1.02 hippocampal seizures during the total kindling procedure, while saline-treated controls experienced on average 3.25 ± 0.85 seizures (Figure 4B). Total seizure duration was significantly higher in animals treated with YIL781 (119.9 ± 14.10 s versus 39.75 ± 10.85 s for saline-treated controls; *p* < 0.05; Figure 4C). In line with these data, average seizure duration was 19.91 ± 1.03 s for YIL781-treated animals compared to an average seizure duration of 12.23 ± 0.74 s for saline-treated controls (*p* < 0.05; Figure 4D). EEG recordings from two saline-treated control animals and from one YIL781-treated mouse were discarded from EEG analyses due to technical reasons.

**Figure 5:**
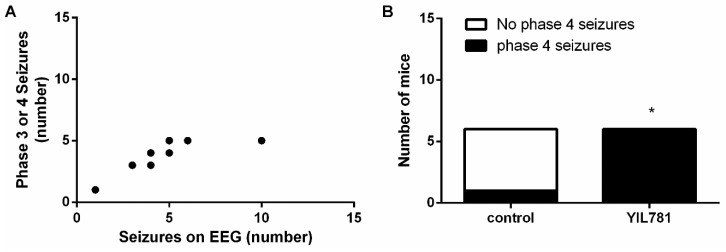
All YIL781-treated mice experience Phase IV seizures. (**A**) Correlation between number of Phase III and IV seizures visually observed and number of hippocampal discharges on EEG (controls and YIL781-treated mice taken together; *n* = 9 pairs). Pearson correlation (*p* < 0.01; Pearson’s r = 0.8113). (**B**) One-hundred percent of YIL781-treated mice (*n* = 6) experienced Phase IV seizures while 17% of saline-treated controls (*n* = 6) did. Fisher’s exact test (*p* < 0.05). * *p* < 0.05; EEG, electro-encephalography.

There was a correlation between Phase III and IV seizures and hippocampal discharges observed on EEG (*p* < 0.01; Figure 5A). Interestingly, 100% of YIL781-treated mice experienced Phase IV seizures during the kindling procedure compared to only 17% of saline-treated controls (*p* < 0.05; Figure 5B). Although this proportion of saline-treated control mice experiencing Phase IV seizures appears to be low, this did not differ significantly from the percentage of control mice experiencing Phase IV seizures in experiment 1 or 3.

### 2.4. The Ghrelin-R Antagonist, JMV-2959, Does not Affect Seizures in the D1R-Mediated Kindling Model

**Figure 6:**
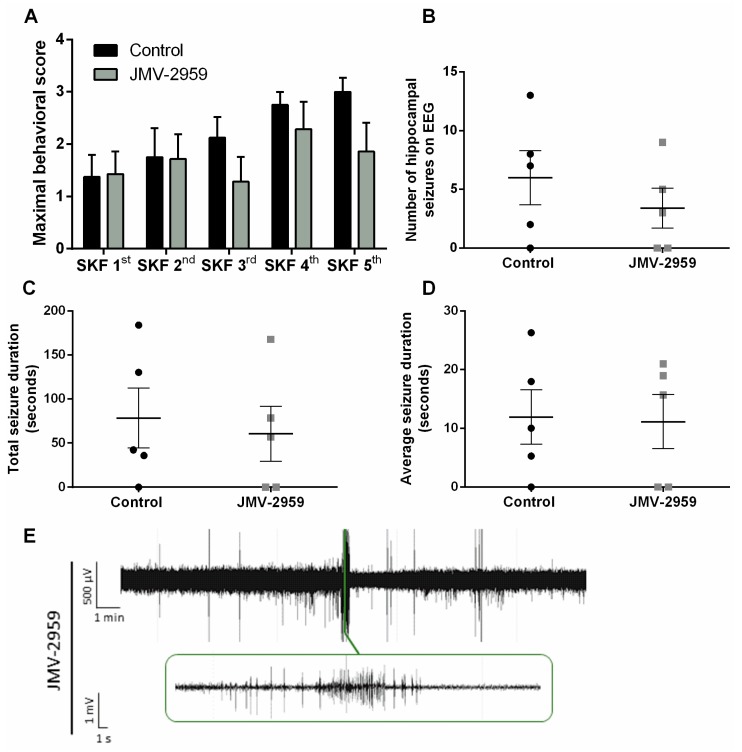
The effect of JMV-2959 on SKF81297 induced seizures. (**A**) Maximal behavioral scores obtained by JMV-2959-treated (*n* = 7) and control (*n* = 8) animals. Two-way RM ANOVA (interaction *p* > 0.05, F(4,52) = 0.8936; time *p* < 0.05, F(4,52) = 3.263; treatment *p* > 0.05, F(1,13) = 1.525). (**B**) The number of hippocampal seizures on EEG did not differ between JMV-2959-treated mice (*n* = 5) and saline-treated control mice (*n* = 5). Mann–Whitney test (*p* > 0.05; Mann–Whitney U = 9.000). (**C**) Total seizure duration did not differ between JMV-2959-treated mice (*n* = 5) and saline-treated control mice (*n* = 5). (**D**) JMV-2959-treated mice (*n* = 5) did not differ in average seizure duration compared to saline-treated control mice (*n* = 5). Mann–Whitney test (*p* < 0.05; Mann–Whitney U = 12.00). (**E**) Representative trace of JMV-2959-treated mouse during SKF 5th. 0.3 Hz high-pass, 60 Hz low-pass, and 50 Hz power line filters were applied. Data are presented as mean ± SEM. EEG, electro-encephalography; µV, microvolt; mV, millivolt; min, minute; s, second; SKF, SKF81297.

In a final experiment, we addressed the effects of the ghrelin-R antagonist JMV-2959 in the D1R-mediated kindling model. This ligand, which does not alter agonist-independent receptor signaling and does not recruit β-arrestin, was administered 30 min prior to kindling and seizure severity and number were assessed. Maximal behavioral score, reflecting seizure severity, did not differ between JMV-2959-treated mice and saline-treated control mice (Figure 6A). JMV-2959-treated mice had an average of 3.4 ± 1.69 hippocampal seizures during the total kindling procedure, which did not differ significantly from the number of seizures that saline-treated control animals experienced (6.0 ± 2.30; Figure 6B). JMV-2959-treated mice had a total seizure duration of 60.62 ± 30.95 s while saline-treated control mice had a total seizure duration of 78.48 ± 33.98 (Figure 6C). Correspondingly, average seizure duration was 11.12 ± 4.62 s for JMV-2959-treated mice compared to an average seizure duration of 11.91 ± 4.65 s for saline-treated control mice (Figure 6D). Also here, sample sizes of mice used for EEG analyses are smaller compared to the number of mice used for behavior as some animals were discarded from EEG analyses due to technical reasons.

**Figure 7:**
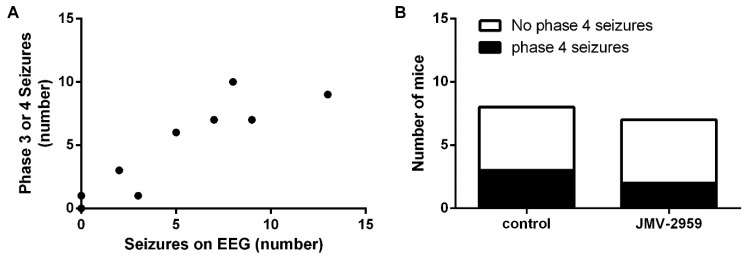
JMV-2959-treated mice experience a similar amount of Phase IV seizures. (**A**) Correlation between number of Phase III and IV seizures visually observed and number of hippocampal discharges on EEG (controls and JMV-2959-treated mice taken together; *n* = 10 pairs). Pearson correlation (*p* < 0.001; Pearson’s r = 0.9144). (**B**) Twenty-nine percent of JMV-2959-treated mice (*n* = 7) experienced Phase IV seizures and 38% of saline-treated controls (*n* = 8) did. Fisher’s exact test (*p* > 0.05). EEG, electro-encephalography.

There was a correlation between the occurrence of Phase IV seizures and hippocampal discharges measured on EEG (*p* < 0.001; Figure 7A). Twenty-nine percent of JMV-2959-treated mice experienced Phase IV seizures during the kindling procedure, which was comparable to 38% of saline-treated controls (Figure 7B).

## 3. Discussion

We demonstrated that the ghrelin-R full agonist, JMV-1843, reduced SKF81297-induced seizure severity and lowered the number of seizures. Additionally, JMV-1843 abolished Phase IV seizures, corresponding to tonic–clonic seizures accompanied with wild jumping, in all animals. These results are consistent with a study demonstrating anticonvulsive effects of JMV-1843 in the 6 Hz acute and kindling model [31], but not with a study that examined JMV-1843 in the rat status epilepticus pilocarpine model [13], albeit at lower doses.

After ghrelin-R agonist binding and G-protein dissociation, β-arrestin is recruited toward the receptor and induces internalization of the receptor via clathrin-mediated mechanisms [23]. This process occurs within minutes of ghrelin stimulation, with a full resensitization taking place after 24 h as was demonstrated in vitro [12,34]. β-arrestin mediates, besides internalization of the ghrelin-R, activation of G-protein-independent signaling. In light of these previous observations, the hypothesis emerged that it is the “inactivation” of the ghrelin-R that was contributing to the anticonvulsive effects mediated by a ghrelin-R full agonist [12].

Therefore, we decided to investigate the effects of the orally available [35] Gα_q/11_ and Gα_12_ ghrelin-R biased agonist YIL781 that is not able to recruit β-arrestin [28], and demonstrated for the first time its effects in a kindling model. We observed that YIL781-treated animals experienced prolonged seizures and had a higher disposition for Phase IV tonic–clonic seizures compared to controls. The inability to internalize ghrelin-R would theoretically lead to sustained Gα_q_ signaling and might be a possible explanation for the enhanced seizure duration and the increased amount of severe seizures that we observed in this model. These observations strengthen our hypothesis that the Gα_q_ downstream signaling pathway is not crucial for ghrelin-R-mediated anticonvulsant actions, but that β-arrestin-mediated internalization or signaling could be pivotal for mediating JMV-1843′s anticonvulsive effects.

In the third experiment, we investigated the effect of a ghrelin-R antagonist, JMV-2959, in the D1R-mediated kindling model. Previous studies demonstrated that JMV-2959 did not exert anticonvulsive effects in the pilocarpine model, in the 6Hz acute model, and in fully kindled mice [14,31]. It was therefore not surprising that also in this model seizure relief was not achieved, as we found no differences regarding seizure severity and number of seizures experienced.

JMV-2959 was recently demonstrated to be partially agonistic toward Gα_q_ in vitro in a ghrelin-R mutated receptor exhibiting low constitutive activity [20], which would render its pharmacological profile to resemble YIL781. However, multiple studies have demonstrated that JMV-2959 inhibits food intake [30,36], while the Gα_q_ proportion of intracellular signaling was recently shown to be responsible for an increase in food intake [28]. Several elements that play a role in these ambiguous observations can be considered, such as ligand behavior depending on receptor constitutive activity [20], or the presence of endogenously available ghrelin. Further studies are highly required to elaborate on a possible biased agonistic character of JMV-2959.

The ghrelin-R is known to form heterodimeric complexes with the D1R, especially prevalent in hippocampus [33], shifting canonical D1R-associated Gα_s_ signaling toward ghrelin-R-mediated Gα_q_ signaling upon stimulation of D1R with SKF81297 [33]. These interactions were shown to be functional both in vitro and in vivo, and the SKF81297-induced intracellular calcium mobilization could be prevented by the administration of JMV-2959 or the inverse agonist L-765867 [33]. Additionally, the improved working memory that was observed upon SKF81297 administration was reversed by JMV-2959 co-administration, thereby demonstrating a dependence of D1R-mediated behaviors on ghrelin-R. As JMV-2959 was ineffective in preventing SKF81297-evoked seizures, we concluded that SKF81297-mediated kindling through D1R activation cannot be blocked by the administration of a ghrelin-R antagonist.

Overall, this study revealed the relevance of biased signaling in ghrelin-R-mediated effects on excitability. The ghrelin-R full agonist, JMV-1843, promoting all ghrelin-R associated downstream signaling pathways and recruiting β-arrestin, was anticonvulsive in the D1R-mediated kindling model. The results obtained from the experiments with the ghrelin-R biased agonist YIL781 suggest that the Gα_q_ and Gα_12_ pathway do not mediate the anticonvulsive effects observed with the full ghrelin-R agonist. Therefore, a role for β-arrestin in desensitization and internalization of the receptor is emerging and is in accordance with previous observations showing that ghrelin-R inverse agonists are anticonvulsant, and that ghrelin-R knock-out mice have increased seizure thresholds [12]. Ideally, a ghrelin-R β-arrestin biased agonist should be developed to get conclusive evidence on whether anticonvulsive effects are mediated by β-arrestin recruitment.

Together, these observations suggest that Gα_q_ or Gα_12_ signaling pathways are not responsible for mediating JMV-1843′s anticonvulsive effects, and encourage further investigations into selective pharmacological intervention of the ghrelin-R in epilepsy models.

## 4. Materials and Methods

### 4.1. Animals

A total of 48 nine to eleven-week-old male C57BL/6J mice (Janvier Laboratories, France) were used in this study. Animals were kept in a 12/12 h light/dark cycle, under temperature (19–23 °C) and humidity (30–70% relative humidity) controlled conditions. They were single housed starting immediately after transmitter and electrode placement, and received regular chow and water ad libitum. All procedures were in accordance with the National Rules on Animal Experimentation and were approved by the Ethical Committee for Animal Experiments of the Faculty of Medicine and Pharmacy of the Vrije Universiteit Brussel, Brussels, Belgium (Ethical approval *n*°: 17-213-2, license date: May 15th 2017). To the best of our understanding, results were described conform the ARRIVE guidelines [37].

### 4.2. Electrode Placement and Transmitter Implantation

A sterilized radiotelemetric mouse transmitter (F10-EET, DSI^®^, Tilburg, The Netherlands) was implanted to monitor EEG changes in mice. Mice were prophylactically treated with enrofloxacin (5 mg/kg) and ketofen (5 mg/kg) and were anesthetized with 3.5 % isoflurane (Vetflurane, Leuven, Belgium) in 100% oxygen and maintained during the entire duration of the surgery by 2.5% isoflurane in 100% oxygen via a facemask. The transmitter was placed i.p., while the recording and reference electrodes were subcutaneously tunneled toward the skull. The peritoneum and skin were closed using non-absorbable suturing material (Ethilon II, 4-0, M-2, Ethicon, Somerville, NJ, USA). The stainless-steel coated, bipolar recording electrode (E363/3/SPC Invivo1) was stereotaxically positioned in the right CA1 region of hippocampus (anterio-posterior: −2 mm; medio-lateral: −1.5 mm; dorso-ventral: −2.1 mm relative to bregma) while the reference electrode was placed at the height of the cerebellum. An anchor screw was placed at the height of the contralateral hippocampus, after which electrodes were fixed to the skull with a smooth layer of dental acrylic cement (Integrity^®^, Denstply Sirona, York, PA, USA). After surgery, mice were placed on a heating pad and were given 0.4 mL 0.9 % NaCl (Baxter, Braine-l’Alleud, Belgium) to prevent post-operative dehydration, and allowed to recover for one week prior to beginning experiments.

### 4.3. Treatment and D1R-Kindling Model

Mice were ad random divided over experimental groups. Mice received one of the following treatments 30 min prior to SKF81297 injection: saline, JMV-1843, JMV-2959 (provided by Prof. Fehrentz), or YIL781 hydrochloride (Tocris, Bristol, UK). All drugs were dissolved in 0.9 % (*w*/*v*) NaCl (5 mg/kg; body volume of 10 mL/kg) and administered i.p. The D1R agonist SKF81297 hydrobromide (5 mg/kg i.p., Tocris) was dissolved in 0.9 % (*w*/*v*) NaCl and administered according to the treatment regimen previously described (Figure 1A) [32].

After SKF81297 administration, animals were evaluated for behavioral seizures during 45 min and assigned a score based on the following observations: 1 = transient disruption of exploratory behavior by tonic immobility/rigidity; 2 = rearing with forepaw myoclonus; 3 = generalized clonus; 4 = tonic–clonic seizure or rapid jumping and wild running [32]. All conducting researchers were blinded for treatment.

### 4.4. EEG Recordings

Freely moving mice were monitored during kindling via EEG registration-units (DSI), consisting of a receiver plate (RPC-1 receiver, DSI), which acquires radio-frequency transmitted signals from the transmitter and sends it to the data exchange matrix (MX2, Matrix 2.0, DSI). EEG signals were sampled at a rate of 500 Hz (Ponemah software, DSI). During EEG analyses with Neuroscore software (DSI), a 0.3 Hz high-pass filter, a 60 Hz low-pass filter, and a 50 Hz notch filter were applied to the recorded traces. Seizures were visually detected and defined as high frequency spikes (>3 Hz) with a duration of at least 3 s, and an increase in baseline amplitude of at least 200%. Not all recordings were suitable for EEG analysis due to EEG contamination by, for instance, heart rate. These recordings were discarded from EEG analyses, explaining the smaller sample sizes for EEG analyses compared to behavioral analyses.

### 4.5. Statistical Analyses

Statistical analyses were performed using Graphpad Prism v6.1. For datasets consisting of multiple groups and time points, two-way ANOVA followed by a Bonferroni post-hoc comparison was performed. For datasets containing multiple groups or time points, one-way ANOVA was performed. For datasets comparing two groups, Mann–Whitney U test was performed. A Fisher’s exact test was used for contingency analyses, and a Pearson correlation test was performed for assessing correlations. For all tests, α was set to 0.05, and P values lower than 0.05 were considered significant. Results are expressed as mean ± SEM.

## Figures and Tables

**Table 1:** Signaling pathways employed by JMV-1843, YIL781, and JMV-2959. An arrow upwards denotes activation of a pathway by binding of a ghrelin-R ligand. A hyphen denotes no alterations in basal signaling levels induced by binding of a ghrelin-R ligand. YIL781 and JMV-2959 are both not able to recruit β-arrestin.

	JMV-1843	YIL781	JMV-2959
**Gα_q/11_**	↑	↑	–
**Gα_i/o_**	↑	–	–
**Gα_12_**	↑	↑	–
**Gα_13_**	↑	–	–
**β-arrestin**	↑	–	–

## Supplementary Materials

Supplementary materials can be found at https://www.mdpi.com/1422-0067/20/10/2480/s1.

## Abbreviations

AC	Adenylyl Cyclase
BBB	Blood–Brain Barrier
cAMP	Cyclic Adenosine Monophosphate
D1R	Dopamine 1 Receptor
EEG	Electro-Encephalography
ERK1/2	Extracellular Signal-Regulated Kinases 1 and 2
GH	Growth Hormone
Ghrelin-R	Ghrelin Receptor
GPCR	G-Protein Coupled Receptor
i.p.	Intraperitoneal
IP3	Inositol 1,4,5-trisphosphate
MAPK	Mitogen-Activated Protein Kinase
min	Minute
µV	Microvolt
mV	Millivolt
PLC	Phospholipase C
PTZ	Pentylenetetrazol
RM	Repeated Measures
s	Second
SEM	Standard Error of the Mean
SKF	SKF81297
SRE	Serum Response Element
