# Supplementary material for: Differential Effects of a Full and Biased Ghrelin Receptor Agonist in a Mouse Kindling Model
Source: Int J Mol Sci. 2019 May 20;20(10):2480. doi: 10.3390/ijms20102480 (PMC6567032; doi:10.3390/ijms20102480)
Supplement: Supplementary file 1 [file ijms-20-02480-s001.pdf]

Supplementary material

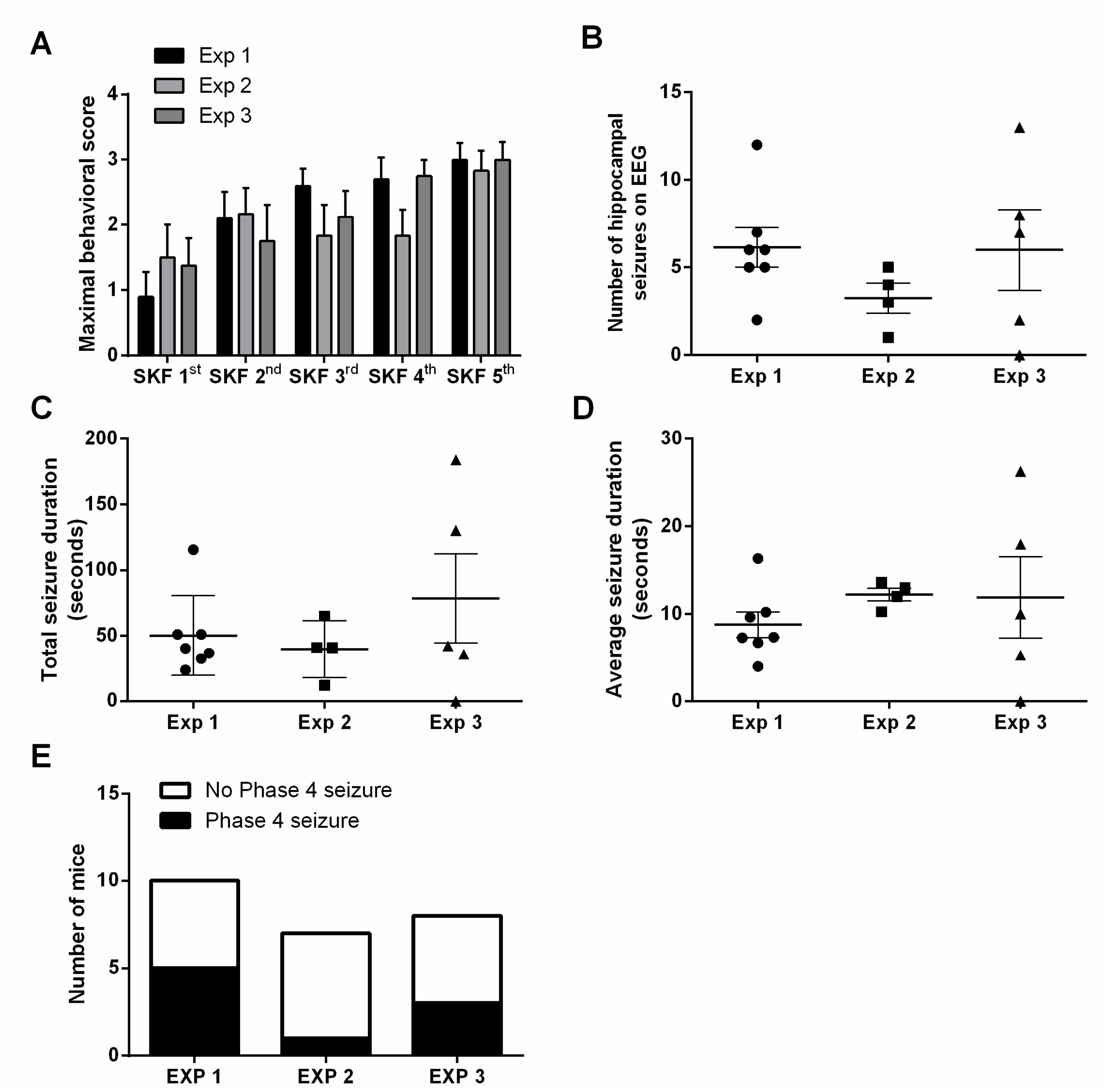

Figure 1: Comparison of the tested parameters between control animals of the three experiments. A) Maximal behavioral scores obtained by saline-treated control animals from the three experiments. Two-Way RM ANOVA (Interaction $P > 0.05$, $F(8, 84) = 0.9064$; Time $P < 0.0001$, $F(4, 84) = 8.408$; Treatment $P > 0.05$, $F(2, 21) = 0.3082$). **B)** The number of hippocampal seizures in saline-treated control animals from the three experiments. Kruskal-Wallis ($P > 0.05$; Kruskal-Wallis statistic = 2.524). **C)** Total seizure duration in saline-treated control animals from the three experiments. Kruskal-Wallis ($P > 0.05$; Kruskal-Wallis statistic = 0.2622). **D)** Average seizure duration in saline-treated control animals from the three experiments. Kruskal-Wallis ($P > 0.05$; Kruskal-Wallis statistic = 2.575). **E)** Proportion of mice experiencing Phase IV seizures during the total kindling procedure Chi-square test ($P > 0.05$; Chi-square 2.291). Data are presented as mean ± SEM. EEG = electro-encephalography; SKF = SKF81297. Exp 1 = control animals from the first experiment (JMV-1843); Exp 2 = control animals from the second experiment (YIL781); Exp 3 = control animals from the third experiment (JMV-2959).

Number of hippocampal seizures on EEG: One-Way ANOVA, $P > 0.05$; Total seizure duration: One-Way ANOVA, $P > 0.05$; Average seizure duration: One-Way ANOVA, $P > 0.05$; Proportion of mice experiencing Phase IV seizures: Fisher's exact test, $P > 0.05$).
